# CANVAR: A Tool for Clinical Annotation of Variants Using ClinVar Databases

**DOI:** 10.1002/mgg3.70020

**Published:** 2024-10-09

**Authors:** Lau K. Vestergaard, Joanna Lopacinska‐Jørgensen, Estrid V. Høgdall

**Affiliations:** ^1^ Molecular Unit, Department of Pathology Herlev Hospital, University of Copenhagen Herlev Denmark

## Abstract

**Background:**

Genomic medicine has transformed clinical genetics by utilizing high‐throughput sequencing technologies to analyze genetic variants associated with diseases. Accurate variant classification is crucial for diagnosis and treatment decisions, and various tools and software such as the Ion Reporter Software and the Illumina Nirvana Software often used in a clinical setting utilize information from the ClinVar database/archive to aid in variant interpretation. However, these existing annotation tools may lack access to the latest ClinVar data, necessitating manual variant inspection.

**Aims:**

To address this gap in developing a tool providing the latest ClinVar data for variant annotation in clinical and research settings.

**Materials and Methods:**

We introduce CANVAR, a Python‐based script that efficiently annotates variants identified from next‐generation sequencing in a clinical or research context, offering comprehensive information from the latest ClinVar database.

**Results:**

CANVAR provides accurate, up‐to‐date variant annotations, streamlining variant analysis.

**Discussion:**

The rise in genomic data requires accurate variant annotation for clinical decision‐making. Misclassification poses risks, and current tools may not always access the latest data, challenging variant interpretation.

**Conclusion:**

CANVAR contributes to enhancing variant annotation by offering comprehensive information from the latest ClinVar database for genetic variants identified through next‐generation sequencing.

## Graphical abstract

Genomic medicine employs DNA high‐throughput sequencing for disease‐related variant analysis. Accurate classification is vital, and outdated information may hinder variant interpretation. Addressing this, CANVAR is introduced, a Python script providing current ClinVar‐based variant annotation to assist variant interpretation.

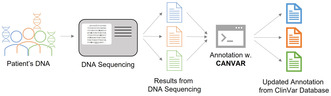

## 1 Introduction

Genomic medicine has revolutionized the field of clinical genetics, offering opportunities for identifying and interpreting genetic variants associated with various diseases (Brittain, Scott, and Thomas 2017). The advent of high‐throughput sequencing technologies and the decreasing cost of genomic sequencing have resulted in a massive influx of genomic data in research and clinical settings (Tromans and Barwell 2022).

Precise annotation enables the classification of variants as benign, likely benign, likely pathogenic, pathogenic, or variants of uncertain significance. Accurate variant classification is essential for providing accurate diagnoses, assessing disease risk, and determining potential treatment strategies. Misclassification of variants might lead to inappropriate medical decisions, potentially endangering patient health and overall well‐being (Samuels, Yu, and Guo 2022; Dashti and Gamieldien 2017).

A wide range of annotation tools and databases have been established in different pipelines to support variant interpretation. ClinVar, a public database, is a central regularly updated repository for variant interpretations submitted by clinical laboratories, researchers, and experts (Landrum et al. 2014; Landrum et al. 2018).

**FIGURE 1:**
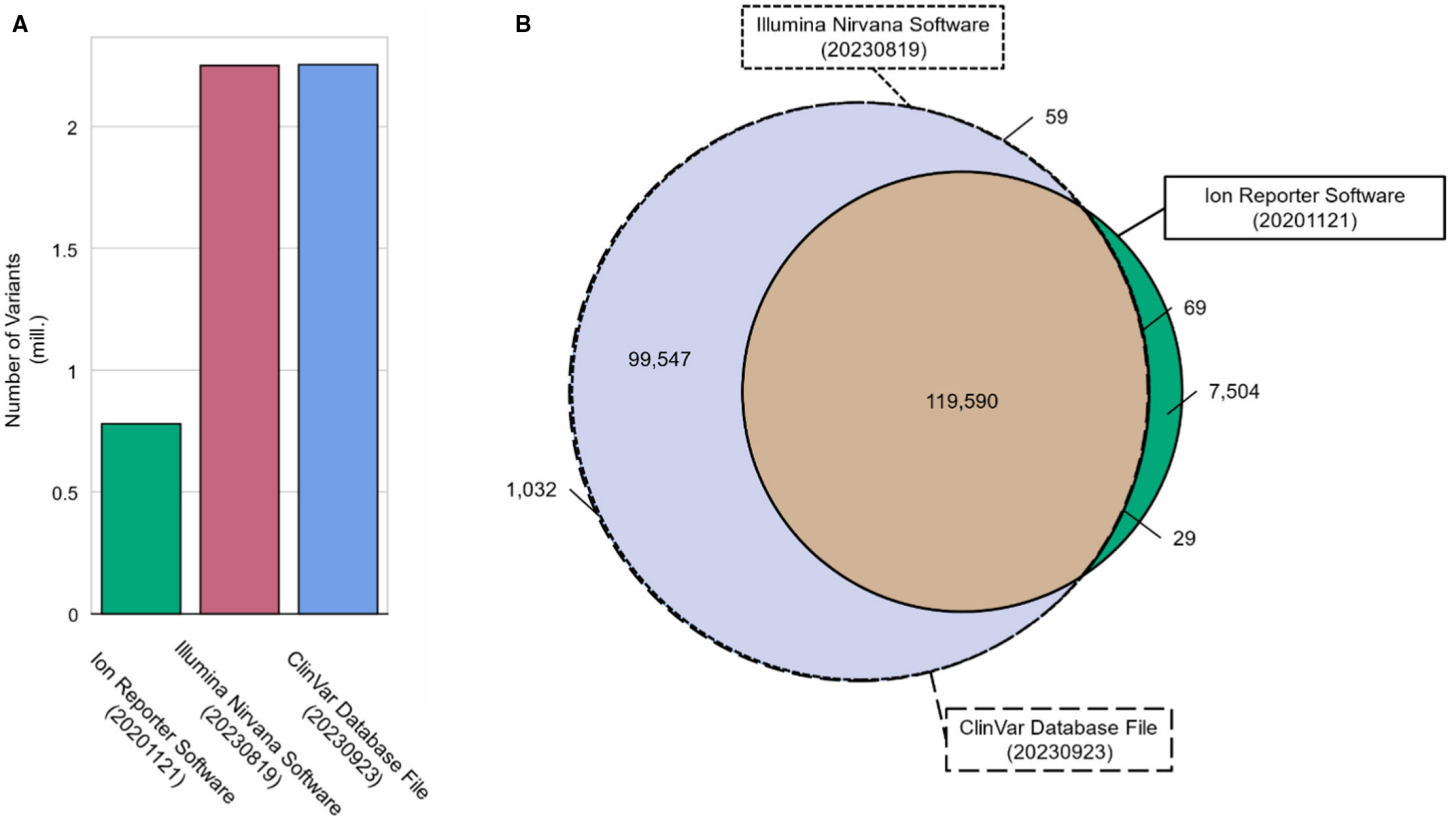
Overview of the number of variants from database files used by the Ion Reporter Software (20201121), the Illumina Nirvana Software (20230819), and the database file from ClinVar (20230923). (A) Number of all variants listed in the database files used by the Ion Reporter Software, the Illumina Nirvana Software, and the database file available from ClinVar (20230923). The annotation file used in the Ion Reporter Software contained 780,733 variants, the Illumina Nirvana Software contained 2,250,892 variants, and the ClinVar database file contained 2,254,400 variants. (B) Venn diagram showing the differences in overlapping and unique variants classified as pathogenic, likely pathogenic, or pathogenic/likely pathogenic between the database files used. The intersection of the three annotation files contained 119,500 variants. The intersection between the Illumnia Nirvana Software and the ClinVar database file contained an additional 99,547 variants. The Ion Reporter contained 7504 unique variants, while the Illumina Nirvana Software contained 59 unique variants, the ClinVar database file contained 1032 unique variants. The Illumina Nirvana Software and the Ion Reporter exhibited an intersection of 69 unique variants. The Ion Reporter Software and the ClinVar database file exhibited 29 unique variants.

Both the Ion Reporter Software from Thermo Fisher and Nirvana, the open‐source software from Illumina, use older versions (20201121 and 20230819, respectively) of variant database files from ClinVar to annotate variants. The ClinVar database file (20230923) contains 2,254,400 variants, while the older versions for the Illumina Nirvana Software contain 2,250,892 variants and the database used by the Ion Reporter Software contains 780,773 variants (Figure 1A). Among the variants classified as pathogenic, likely pathogenic, or pathogenic/likely pathogenic, 119,590 variants are common to both software systems and the ClinVar database file (Figure 1B). The Illumina Nirvana Software and the ClinVar Database file (20230923) have an additional 99,547 variants beyond the intersection, while 59 variants were unique to the Illumina Nirvana Software. The Ion Reporter Software database file harbors 7504 unique variants and shares 69 variants with the Illumina Nirvana Software. The ClinVar database file contains a further 1032 variants and shares 29 variants with the Ion Reporter Software classified as pathogenic, likely pathogenic, or pathogenic/likely pathogenic (Figure 1B).

The ClinVar database is a useful tool for obtaining the latest annotations. There is currently a lack of freely available, user‐friendly tools that can be used to annotate sequencing outputs with the latest ClinVar annotations in a quick and efficient manner. This means that manual inspection of variants may be required, which can be time‐consuming and error‐prone. Additionally, implementation of variants in clinical routine testing can be difficult due to commercialized annotation tools, online tools that require registration, or batch processing that may not be possible. To address these challenges, we present CANVAR, a Python‐based script that efficiently and accurately annotates variants in a clinical context. CANVAR provides contextual information and functional insights to identified variants from next‐generation sequencing, including clinical significance, disease associations, and clinical review status, all directly from the latest ClinVar database.

Updated ClinVar data may significantly contribute a clinical pipeline, by providing the latest variant information from the scientific and medical community. Incorporating newly added or revised variant information may also necessitate the recalibration of current pipelines to ensure precise annotation. This process may involve updating reference databases for standardized tools in a given clinical setting.

**FIGURE 2:**
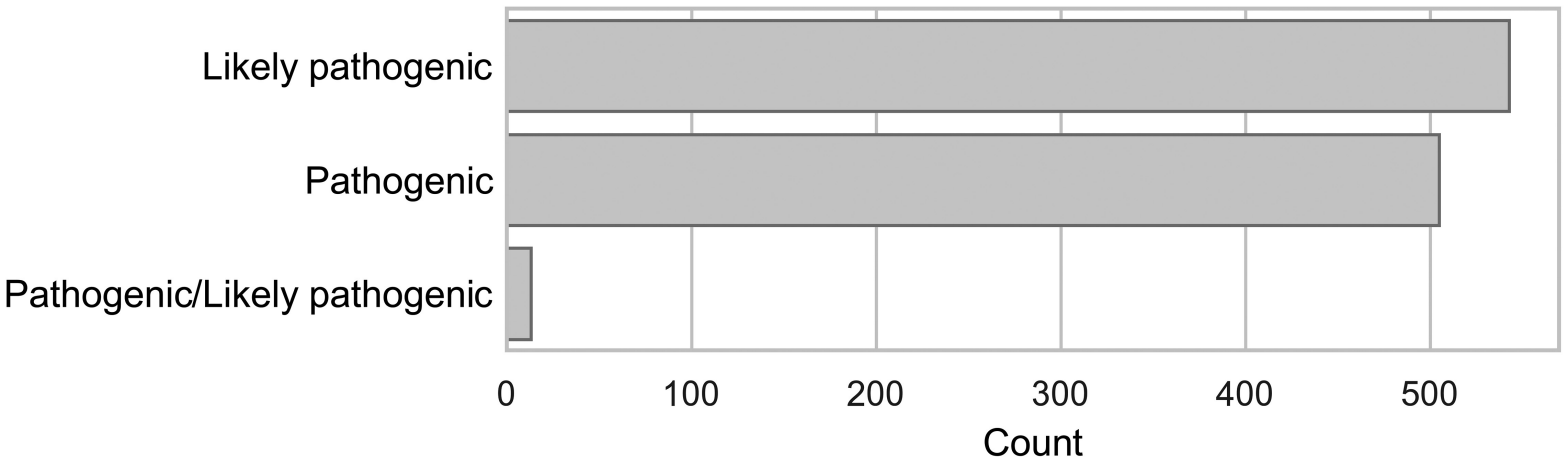
Overview of the distribution of the non‐overlapping variants classified as likely pathogenic, pathogenic, or Pathogenic/likely pathogenic between the Nirvana Software (20230819) and the ClinVar database file (20230923).

The non‐overlap between ClinVar datasets may be attributed to various factors that reflect ongoing updates and modifications in the data over time. ClinVar continuously revises its database as new information and novel variants emerge (Landrum et al. 2020). Existing data can be re‐evaluated, leading to the potential re‐classification of variants based upon updated scientific research or clinical findings (Sharo et al. 2023). Consequently, entries present in older datasets may be absent in newer versions if they have been deemed outdated or incorrect or novel variants are introduced in the newer versions of the database. An example of this is observed in the non‐overlapping variants between the Nirvana Software (20230819) and the newer updated ClinVar database file (20230923). Here, the latter contained an additional 1032 variants that were classified as likely pathogenic, pathogenic, or pathogenic/ likely pathogenic (Figure 2). Thus, it is important to keep and maintain updated versions of database files for variant interpretation.

## 2 Availability and Implementation

CANVAR is freely available, and the source code has been deposited on the GitHub repository (https://github.com/kraesing/CANVAR) CANVAR is written in Python (v.3.7.6) and can be used via the command line. A test file for implementation of the software is available on the GitHub repository.

### 2.1 Packages

The *packages* function installs and imports the necessary packages for CANVAR to function correctly and can be initiated using:python ~/CANVAR.py packages --import_packages Y


Each function has a help option attached and can be accessed for further guidance by calling either:python ~/CANVAR.py --help or CANVAR.py “function” --help


### 2.2 Prearrange

The *prearrange* function creates the environment containing the parent directory ~/canvar and the subdirectories: ~/canvar/archive, ~/canvar/clinvar_database_files, ~/canvar/input_files, ~/canvar/output_files_annotated. The environment and subdirectories are created using the following:python ~/CANVAR.py prearrange --wrkdir ~/path/to/dir


Note that after the execution of the above command line operation, the working directory must be changed to ~/canvar.

### 2.3 Download_db

The *download_db* function connects to the File Transfer Protocol provided by ClinVar, and the latest version of the database can be downloaded using:python ~/CANVAR.py download_db --latest_file latest


Specification of Genome Reference Consortium Human assembly is provided in an interface command line afterward, where either 37 or 38 are specified via the command line.

Options for choosing an earlier database version are also available, and this is achieved using the ‐a argument followed by the year of choice. A list of database files from ClinVar in the specified year will show, and the desired file can be selected by entering the index. Previous database files can be downloaded using the following:python ~/CANVAR.py download_db --archive_file 2021


Database files will be downloaded to ~/canvar/clinvar_database_files.

### 2.4 Check_construct

The *check_construct* function uses the downloaded database file from ClinVar to generate the file needed for annotating variants. Once generated, the annotation file contains information about *location, nucleotide change, gene symbol, clinical significance, reference SNP cluster ID (RS id), mutation type, ClinVar review status, and ClinVar disease name*. This information will be used to annotate variants within the user‐provided input file. The annotation file can be constructed using the .gz format or from the .vcf if the file has been unpacked, using the following:python ~/CANVAR.py check_construct --database_file clinvar_20230617.vcf.gzpython ~/CANVAR.py check_construct --database_file clinvar_20230617.vcf


### 2.5 Annotate

The *annotate* function uses the annotation file created via *check_construct*, and the annotation file used for annotation of variants from user input files is specified using the *‐f* argument. The annotation of user input files is linked to the directory ~/canvar/input_files. The user input files must contain variables for locus position, reference allele, and observed allele, under the specified variable names: *Locus*, *Ref*, and *Observed Allele*. An example of the file format for the user input file is shown in Table 1.

**TABLE 1:** Example of the format for an input file for annotation. The columns: Locus, Ref, and Observed allele must be present under these specified names.

Locus	Ref	Observed allele
chr1:11168337	T	C
chr1:11168338	C	A
chr1:11168338	C	G
chr1:11169361	C	G
chr1:120458435	T	TG
chr1:27105553	C	T
chr7:6026775	T	C
chr9:139410437	C	T
chr13:32929387	T	C
chr17:7578461	C	T

The annotation can be executed using the following:python ~/CANVAR.py annotate --annotation_file clinvar_20230617.tsv


After annotation, the input file has been extended with the following columns *Identifier*, *Gene_symbol*, *Clinical_significance*, *RS_id*, *Mutation_type*, *ClinVar_review_status*, and *ClinVar_disease_name*. If the variant is unknown in the ClinVar database, the variant is flagged with *Manually inspection needed* in the column named *Clinical_significance*. After the annotation process, the original files are moved to ~/canvar/archive, and the annotated (.ann) user files are moved to ~/canvar/output_files_annotated. An example of the output following annotation with CANVAR of the variants outlines in Table 1 is provided in the Data S1.

## 3 Discussion and Conclusion

In the era of genomic medicine, variant annotation has become essential for accurate genetic variant interpretation. The increase in genomic data, driven by advanced sequencing technologies, necessitates precise classification of variants to guide clinical decisions. Misclassification might pose risks to patient well‐being. For this purpose, CANVAR efficiently annotates clinically relevant details from the most recently updated ClinVar database, aiding clinicians in prioritizing variants being relevant for potential precision medicine.

## Author Contributions

L.K.V. contributed to the conceptualization, programming, original manuscript writing, reviewing, and editing. J.L.‐J. and E.V.H. contributed to the conceptualization, writing, reviewing, and editing.

## Conflicts of Interest

The authors declare no conflicts of interest.

## Supporting information


**Data S1.** Supporting Information.

## Data Availability

The data that support the findings of this study are available in CANVAR at https://github.com/kraesing/CANVAR. These data were derived from the following resources available in the public domain: —Index of /pub/clinvar/vcf_GRCh37, https://ftp.ncbi.nlm.nih.gov/pub/clinvar/vcf_GRCh37/—Index of /pub/clinvar/vcf_GRCh38, https://ftp.ncbi.nlm.nih.gov/pub/clinvar/vcf_GRCh38/. Availability and Implementation: CANVAR and Data S1 are available at https://github.com/kraesing/CANVAR under the MIT License terms.
